# Osseointegration in additive-manufactured titanium implants: A systematic review *of animal studies* on the need for surface treatment

**DOI:** 10.1016/j.heliyon.2023.e17105

**Published:** 2023-06-10

**Authors:** João Vicente Calazans Neto, Andréa Cândido dos Reis, Mariana Lima da Costa Valente

**Affiliations:** Department of Dental Materials and Prosthodontics, Ribeirão Preto Dental School, University of São Paulo (USP), Ribeirão Preto, Brazil

## Abstract

The objective of the systematic review is to find an answer to a question: “Do surface treatments on titanium implants produced by additive manufacturing improve osseointegration, compared to untreated surfaces?“. This review followed the Preferred Reporting Items for Systematic Review and Meta-Analysis (PRISMA 2020) and was registered in the International Prospective Register of Systematic Reviews (PROSPERO) (CRD42022321351). Searches were performed in PubMed, Scopus, Science Direct, Embase, and Google Scholar databases on March 22nd, 2022. Articles were chosen in 2 steps by 2 blinded reviewers based on previously selected inclusion criteria: articles in animals that addressed the influence of surface treatments on osseointegration in implants produced by additive manufacturing. Articles were excluded that (1) did not use titanium surface, 2) that did not evaluate surface treatments, 3) that did not described osseointegration, 4) Studies with only in vitro analyses, clinical studies, systematic reviews, book chapters, short communications, conference abstracts, case reports and personal opinions.). 1003 articles were found and, after applying the eligibility criteria, 17 were used for the construction of this review. All included studies found positive osseointegration results from performing surface treatments on titanium. The risk of bias was analyzed using the SYRCLE assessment tool. Surface treatments are proposed to promote changes in the microstructure and composition of the implant surface to favor the adhesion of bone cells responsible for osseointegration. It is observed that despite the benefits generated by the additive manufacturing process in the microstructure of the implant surface, surface treatments are still indispensable, as they can promote more suitable characteristics for bone-implant integration. It can be concluded that the surface treatments evaluated in this systematic review, performed on implants produced by additive manufacturing, optimize osseointegration, as it allows the creation of a micro-nano-textured structure that makes the surface more hydrophilic and allows better contact bone-implant.

## 1 Introduction

The additive manufacturing (AM), like software aid (CAD), technique in the manufacture of implants can allow the manufacture of customized three-dimensional (3D) metallic devices, with controlled surface characteristics, custom geometries and different levels of porosity [[1], [2], [3], [4]]. This justifies the investment to increase the use of this technique in the manufacture of implant parts that require more complex structures, as rougher surfaces, adequate rigidity and good stress transfer [5].

Additive manufacturing in metallic implants is performed by selective laser fusion (SLM) and electron beam fusion (EBM) processors [1,[6], [7], [8]]. Both are made using rapid prototyping technology, where the metal alloy powder is melted by exposing the SLM or EBM process to reproduce, the final model of the implant [1,[9], [10], [11]]. The SLM uses a high power laser beam, for the melting of the metal alloy powder, simultaneously with this melting process, it is released an inert gas in an attempt to protect the particles from overheating [1,5,7].The EBM performs the melting by an electron beam that generates a lower temperature than the SLM, in addition, this process is carried out in a closed chamber, with high vacuum levels, protecting materials from oxidation [1,7].

These methods can reduce the waste of material, time, and money compared to the conventional machining technique, in addition, they aim to create varied structures, with uniform density distribution and control of the quality and quantity of pores on the surface [4,9,10]. This last advantage in AM, since these pores are determinant for the mechanical properties and biological that precede the osseointegration [9,12,13].

It is known, however, that surface treatment techniques, such as TiO2 nanotubes, hydroxyapatite, collagen type I, calcium phosphate, tantalum, polydopamine, acid attack and anodic oxidation, Hydrofluoric/nitric acid, inorganic chemical oxidation, sodium hydroxide, chitosan and UV photofunctionalization, despite their advantages, deserve care, mainly due to the high degree of contamination and toxic elements that can be introduced into titanium, which goes against the principle that the ideal surface for better mechanical stability and osseointegration capacity is the one with the highest purity of titanium oxide and adequate roughness, which thus favor the activities of osteogenic cells and absorption of plasma proteins that regulate osteoblastic adhesion in the biomaterial and thus configure themselves as fundamental characteristics for the osseointegrative process [10,14,15].

Although studies suggest better biological in dental implants obtained by AM [1,3,6,8,16,17]. While articles in the literature evaluate surface properties related to the application of the coating, the originality and relevance of the present systematic review is to evaluate how this surface treatment, whether chemical, electrochemical, or mechanical, influences osseointegration and to understand how this reflects in the best possible way. Bone-implant contact and the absorption of cells that are optimizing this process. Thus, the objective of this systematic review was to present the current state of the art on the need to carry out surface treatments in titanium dental implants produced by additive manufacturing technique. For this, this review was built from the null hypothesis that the performance of surface treatment on titanium dental implants does not influence osseointegration.

## 2 Material and methods

### 2.1 Protocol

The present systematic review sought to answer the question: “Do surface treatments on titanium implants produced by additive manufacturing improve osseointegration, compared to untreated surfaces?” This review was constructed following the preferred reporting items for the systematic review and meta-analysis (PRISMA 2020) [18]. In addition, the protocol was registered in the International Prospective Register of Systematic Reviews (PROSPERO) (CRD42022321351). The search strategy used for a population, intervention, comparison, outcome, and study design (PICOS) is described in Table 1.

**Table 1:** Population/Animals, Intervention, Comparison, Outcome, and Study Design (PICOS) strategy for systematic review.

PICOS	Description
Population/Animals	Titanium surfaces produced by additive manufacturing/The animals involved in the included studies were: rats, rabbits, dogs and sheeps, of both sexes, of different ages and weights.
Intervention	Surface treatments: TiO2 nanotubes, hydroxyapatite, collagen type I, calcium phosphate, tantalum, polydopamine, acid attack and anodic oxidation, Hydrofluoric/nitric acid, inorganic chemical oxidation, sodium hydroxide, chitosan, and UV photofunctionalization.
Comparison	Control Group (additive manufactured titanium surfaces that have not received surface treatments)
Outcome	Osseointegration was assessed using techniques such as micro computed tomography, histological imaging, and MICRO-CT analysis.
Study Design	In vivo studies

PICOS, Population, Intervention, Comparison, Outcome and Study Design.

### 2.2 Eligibility criteria

This review included articles that addressed the influence of surface treatments on osseointegration in manufactured implants. For that, articles with in vivo analyses in animals were used. After defining the exclusion criteria, articles that did not use titanium surfaces or that used titanium surfaces that were not produced by additive manufacturing, that did not evaluate surface treatments and that did not describe osseointegration were removed. Studies with only in vitro analyses, clinical studies, systematic reviews, book chapters, short communications, conference abstracts, case reports, and personal opinions were also excluded.

### 2.3 Search strategy

The search strategy was applied to the following electronic databases: PubMed, Scopus, Science Direct, Embase, and Google Scholar (Supplementary Table 1) *in March 22nd, 2022*. This search was performed and the selected studies, based on the eligibility criteria, are published between the years 2015–2022. In addition, an additional search was performed in the reference and citation lists of the included articles to find new possible inclusions.

The first reading of the articles was performed by 1 author (J.V·C·N.). The findings were attached to the Rayyan digital platform and then evaluated by 2 independent authors (J.V·C·N., M.L.C·V.) who were responsible for analyzing the articles according to the pre-established inclusion and exclusion criteria. The other studies were read in full. Conflicting results were resolved by the third author (A.C.R.). Data extraction from the article was done through a table with the following topics: Author/year; Goal; Evaluated Animals; Titanium Alloy; Type of Coating; Control Group Surface; Test Group Surface; Assessment Method; Conclusion.

### 2.4 Risk of bias assessment

For this evaluation, the tool for animal studies called SYRCLE [19] was used (Supplementary Table 2). Based on the analysis of these criteria, the article is classified as having a low, high, or uncertain risk of bias. When using this tool, it is not recommended to perform calculations and score charts per study.

From the application of the SYRCLE tool, it was observed that in all articles included there were: similar basic characteristics between the groups; concealed allocation to different groups during the study; the case and control groups were randomly distributed among the housing being exposed to the same conditions; random assessment of the outcome of the case and control groups; all results were treated completely; protocol availability and reporting of all results; no other sources of bias were presented.

### 2.5 Certainty of the evidence

The certainty of the evidence for each outcome was calculated by the Grading of Recommendations Assessment, Development, and Evaluation (GRADE) approach. In this assessment, two independent authors (J.V·C.N. and M.L.C·V.) analyzed the certainty of evidence. Initially, the results are considered with high-quality evidence and the downgrade of the evidence is given by limitations, inconsistencies, indirectness, imprecision, and publication bias criteria. Disagreements were resolved by consensus. Based on the mentioned criteria, the certainty of each piece of evidence was classified as high, moderate, low, and very low [20].

## 3 Results

### 3.1 Search results

**Fig. 1:**
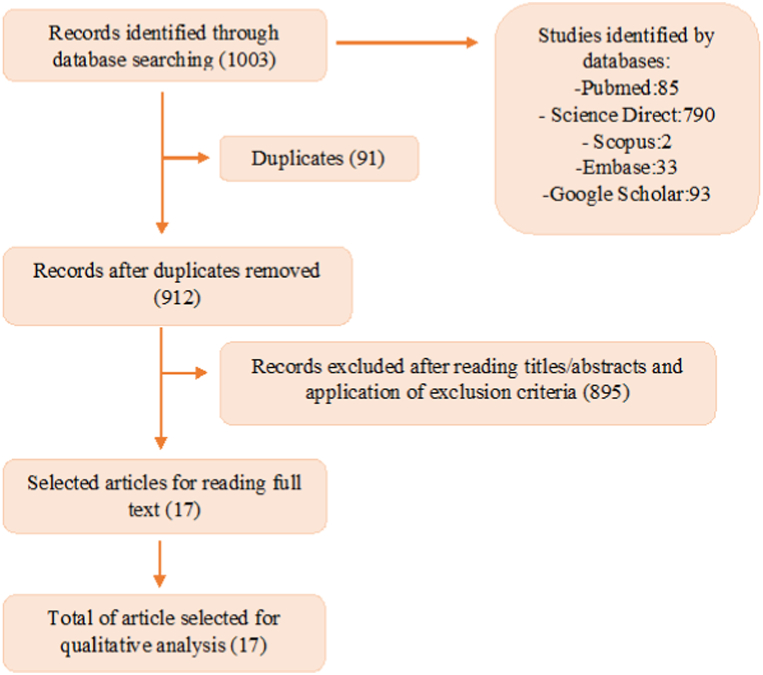
Flow diagram summarizing selection process.

Fig. 1 addresses the strategy used to select the studies. In the first search, 1003 articles were found, of which 91 were excluded due to duplication. After reading the title and abstract with the application of inclusion and exclusion criteria, 17 studies were selected for a full reading. After reading in full, the 17 articles were included for analysis and discussion in this review.

Performing a meta-analysis was not possible due to the lack of homogeneity of results found in the included articles. Thus, the results were based on a descriptive analysis of the data.

### 3.2 Risk of bias assessment

In line with the risk of bias by the SYRCLE, the studies by Gu et al. [21], Lee et al. [4], Li et al. [11], Mitra et al. [22], Ren et al. [23], Wang et al. [24] and Wang et al. [25] did not explain whether the allocation sequence was correctly generated and applied between the evaluated groups (if and control). While in the other studies it was exposed that the case and control groups were correctly allocated randomly [5,9,[26], [27], [28], [29], [30], [31], [32], [33], [34]].

In addition, the studies described that researchers were not aware of which animals received each type of intervention [4,9,11,[21], [22], [23],[26], [27], [28]], except Bandyopadhyay et al. [26], Teng et al. [30], Wang et al. [12], Wang et al. [25], Xu et al. [5], and Yavari et al. [33], who did not make it clear whether the investigators were blinded to the knowledge of which intervention each animal received during the experiment. Still regarding the blinding of the evaluators during the research, the authors Bandyopadhyay et al. [26], Teng et al. [30], Wang et al. [12], Wang et al. [25], Xu et al. [5], and Yavari et al. [33], did not describe whether the researcher was aware when evaluating the outcome, about which animals had received each type of intervention.

### 3.3 Grading the evidence

For the certainty of the evidence of the selected studies, the GRADE tool was applied. The studies included in this review are randomized pre-clinical studies, the evaluation of which is initiated at a high level of evidence, and as the gradation of quality is performed, this level can be lowered. However, the factors involved in the quality of evidence, such as the risk of bias, inconsistencies, indirect evidence, and imprecision in the results, maintained the certainty of the evidence of the results found at a high level. (Supplementary Table 3).

### 3.4 Characteristics of the studies

Table 2 provides information regarding the included studies, based on the eligibility criteria, which are published in the years 2015–2022.

**Table 2:** Provides information regarding the included studies, which were published between 2015 and 2021.

Author/year	Objective	Evaluated Animals	Titanium Alloy	Type of Coating	Control Group Surface	Surface Test Group	Assessment Method	Conclusion
Bandyopadhyay et al., 2016^23^	To assess whether the porosity of manufactured implants can optimize osseointegration.	Twelve male Sprague-Dawley rats weighing between 280 and 300 g.	Commercially pure Titanium.	TiO2 nanotubes.	Dense pure Ti	Porous Ti (Laser Engiered Net Shapg (LENSTM)) with nanotubes.	Computerized Microtomography; Histological image; Scanning Electron Microscopy; Mechanical push-out tests.	Implants manufactured from titanium, with or without surface modification, improve the initial stage of osseointegration.
Brogini et al., 2021^9^	To investigate osseointegration in implants manufactured from Ti–6Al–4V, with or without post-processing of HA and collagen type I in an in vivo model.	Rabbits.	Ti–6Al– 4 V.	Hydroxyapatite (Ti-POR + HA) and collagen type I (Ti-POR-COLL).	Ti–6Al–4V interconnected porous structure, with a solid central core and a porous outer layer.	Ti–6Al–4V + 50 ÿm thick layer of HA; Ti–6Al–4V + type I equine collagen.	Histological, histomorphometric and microhardness evaluations.	Manufactured materials optimize osseointegration and allow functionalization of hydroxyapatite or Type I collagen to support long-term clinical outcomes.
Gu et al., 2022^18^	Observe whether the surface treatment is beneficial for new bone formation after four weeks.	Six adult male beagle dogs.	Ti–6Al–4V.	Biomimetic coating of CaP.	Ti6Al4V without any coating	Ti6Al4V with biomimetic coating.	Compression tests, Scanning Electron Microscopy; Micro-CT analysis; Histological and histomorphometric analyses.	Coatings create a new surface that can induce better osseointegration.
Huang et al., 2020^24^	To assess whether manufactured implants surface-treated with calcium phosphate improves osseointegration.	Twenty-seven New Zealand White Rabbits.	Ti–6Al–4V.	Calcium phosphate.	Traditionally manufactured titanium screw.	Porous screw with bioceramic coating.	Biomechanical analyses; Histological analyses. Imaging exams.	Implant screws manufactured with bioactive surface modification improved osseointegration.
Lee et al., 2021^4^	Analyze the result of implants printed without surface treatment, with SLA and with TIPS surface treatment.	Twenty-seven rabbits weighing between 3 and 4 kg.	Commercially available grade 2 titanium.	Tantalum.	Implant manufactured without surface treatment.	implant manufactured with ion-induced treatment.	Histological and histomorphometric analyzes.	Treating the surface with TIPS on manufactured implants can improve the osseointegration phenomenon.
Li et al., 2015^11^	To evaluate whether polydopamine (pDA) and hydroxyapatite coating on manufactured implant surfaces optimize osseointegration and osteogenesis.	Eighteen male New Zealand white rabbits weighing 2.5–3.5 kg.	Ti6Al4V.	Hydroxyapatite (HA)/Polydopamine (pDA).	Ti6Al4V uncoated.	Ti6Al4V coated with pDA by immersion.	Field emission scanning electron microscopy; Element dispersive energy spectroscopic (EDS); Real-time quantitative PCR (RT-PCR); Micro-CT; Histological analysis.	Surface-modified Ti6Al4V are attractive for use as bone substitutes in orthopedic applications.
Mitra et al., 2021^19^	Increase biocompatibility and osseointegration of imprinted implants through a chemical combination of the tantalum (Ta) surface.	Male Sprague-Dawley rats weighing between 280 and 300 g; New Zealand male white rabbits with weight between 3.5 and 4 kg.	CpTi-Ta.	Tântalo (Ta).	Ti6AL4V.	Ti6AL4V with surface treatment (CpTi-Ta)	Field Emission Scanning Electron; Vickers Microhardness; Compression tests; Micro-CT analysis; Biological and histomorphometric analysis; energy dispersive x-rays (EDS) and FESEM analysis.	Porous Ti–Ta alloys increase tissue creation at the bone-implant interface, which demonstrates a superior biological result of the material compared to the initial stage bone healing.
Ren et al., 2020^20^	Optimize the biological activity and osteogenesis of Implants manufactured using a hierarchical micro/nanotopography on the surface.	Adult male rats Sprague Dawley (SD) at about 6 weeks and 190–200 g.	Ti6Al4V.	Acid attack and anodic oxidation (AN).	Ti6Al4V polished.	Ti6Al4V coated by anodic oxidation.	Scanning electron microscope; Wetability; SBF immersion test; Micro-CT X-ray Energy Dispersion Spectroscopy (EDS); Histological analysis.	The surface treatment improves the bioactivity and osteogenic properties of the manufactured implant, accelerating osseointegration.
Rony et al., 2021^25^	To evaluate the in vivo osseointegration of two types of titanium cylinders.	18 non-GMO sheep purchased from local breeders (France).	Ti6Al4V.	Hydrofluoric/nitric acid solution (HF/HNO3).	Filled with titanium geometric cylinder.	Filled with a titanium trabecular cylinder.	Scanning Electron Microscopy; Micro-CT analysis; Computed tomography; Histological and histomorphometric analysis.	Implants manufactured from coated titanium provide osseointegration over time. However, it can be seen that the treatment is more effective on the outermost surfaces and does not seem to reach the central cavities of the samples.
Shu et al., 2021^26^	To evaluate osseointegration in implants manufactured with micro-nanotopography by means of inorganic chemical oxidation.	New Zealand male rabbits with an average weight of 3.5 kg.	Ti6Al4V.	Inorganic chemical oxidation.	Ti6Al4V.	Ti6Al4V with chemical oxidation surface.	FE-SEM; EDS, Profilometer; AFM; Contact angle meter; qRT-PCR; Specimen collection; Micro-CT analysis.	Micro-nanotopography by means of inorganic chemical oxidation increases osteoblastic activity and thus can optimize osseointegration.
Teng et al., 2019^27^	Observe if the surface modification influences the success of the implant.	Adult white New Zealand rabbits weighing 3.5–4.5 kg.	Ti6Al4V.	Ca–P by the microarc oxidation (MAO) method.	Ti6Al4V with 600 ÿm pore size by 3D printing.	Ca–P coating was deposited on MAO treated porous Ti alloys.	Twist test; Micro-CT analysis; Scanning electron microscopy; Energy dispersive spectroscopy.	The coated implant had its fixation capacity improved, in addition, the appearance of blood vessels in the central region of the implant was noticed.
Wang et al., 2016^21^	To evaluate whether coating implants manufactured with tantalum through chemical vapor deposition enhances osseointegration.	Six diabetic sheep.	Titânio poroso (TiI).	Tantalum.	Porous titanium (TiI).	Porous titanium (TiI) coated with tantalum by chemical vapor.	Morphological analysis; Western Blot analysis; Micro-CT analysis; Analysis of histology and histomorphometry.	The antioxidant characteristics of this coating can accelerate osseointegration and reduce clinical implant failure.
Wang et al., 2018 ^12^	To assess whether nanotopographic modifications in manufactured implants improve osteogenic differentiation and osseointegration.	Ten female SD Rats weighing around 280 g.	Ti6Al4V.	NaOH solution.	Ti6Al4V porous 3D printing.	Ti6Al4V porous 3D printed immersed in NaOH solution.	Scanning electron microscope; X-ray diffractometer; Wetability; X-rays with energy dispersion spectrometry; Alkaline Phosphate Activity Assay; qRT-PCR; Alizarin Red A Staining Assay; Histomorphometric Analysis;	Surface modification in manufactured implants favors osseointegration in the early stages. It is noticed that these implants can replace conventional implants.
Xiu et al., 2016^29^	To investigate whether surface treatment by microarc oxidation on 3D-printed implants improves osseointegration.	Twenty-seven male New Zealand rabbits.	Ti6Al4V.	Stepped Micro-Arc Oxidation (MAO).	Porous Ti6Al4V.	Porous Ti6Al4V with MAO process.	Scanning Electronic Microscope; X-ray photoelectron spectroscopy; Micro-CT analysis; Histological analysis; Push test.	A faster and longer lasting osseointegration occurred in the coated implants.
Xu et al., 2016^5^	To evaluate the performance in bone regeneration on implant surfaces with topographical modification.	12 New Zealand white rabbits weighing 2.0–2.5 k.	Commercially pure grade II titanium.	40% HF and 60% HNO3 (H2O/HF/HNO3, 1:4:5, v:v); 98% H2SO4 and 36.5% HCl solution (H2O/HCl/H2SO4, 2:4:3, v:v).	Pure grade II titanium.	Pure grade II titanium blasted with 250 μm particles of ZrO2 and then immersed in an acid mixture consisting of 40% HF and 60% HNO3 (H2O/HF/HNO3, 1:4:5, v:v).	Field emission scanning electron microscopy; X-ray photoelectron spectroscopy; Analysis of roughness and contact angle; Assessment of cell viability; Micro-CT analysis; Biomechanical tests.	Surface treatment accelerated bone growth and increased contact at the bone-implant interface in vivo.
Yavari et al., 2019^30^	Developing surface coatings by the layer-by-layer technique to analyze their ability to prevent infections and stimulate bone regeneration.	Five male Fisher rats at 16 weeks of age.	*C*P-Ti (grade 1).	Chitosan and gelatins with cationic and anionic bases.	Porous titanium discs.	Porous titanium discs with 5 empty gelatin/chitosan bilayers were loaded.	Micro-CT; Scanning electron microscope; Photoelectron Spectroscopy (XPS); Cell viability assays; Histological analysis.	Surface biofunctionalization has the ability to prevent infections and stimulate bone tissue regeneration.
Yin et al., 2021^31^	To evaluate ultraviolet (UV) photofunctionalization on surface surfaces of manufactured implants and its effects on osseointegration.	Twenty-seven adult male New Zea rabbits.	Ti6Al4V.	UV photofunctionalization.	Ti6Al4V.	Ti6Al4V with UV treatment.	Wetting measurements; Field emission scanning electron microscopy; X-ray photoelectron spectroscopy; Alkaline phosphatase activity; Mechanical tests; Micro-CT analysis; Histological examination; Push test.	The present surface treatment method can improve osseointegration in manufactured implants while preserving their macromorphology.

Bandyopadhyay et al. [26] found that implant biocompatibility was increased and thus osseointegration was optimized, although the authors also support the use of an uncoated manufactured implant [26]. Gu et al. [21], Wang et al. [25], Teng et al. [30] and Yavari et al. [33] found better osteoblast induction, differentiation of osteogenic cells that induce osseointegration and accelerated healing [21,25,33,34]. Huang et al. [27] found that the coating improves osseointegration in the early stages of implant placement [27]. Lee et al. [4], Wang et al. [12], Mitra et al. [22], Ren et al. [23], Brogini et al. [9], Li et al. [11] and Rony et al. [28] proved a promotion in the osseointegration characteristics of the implant [4,9,11,12,22,23,28], however, Lee et al. [4] add that after 12 weeks the implants manufactured uncoated achieve the same level of osseointegration [4].

Shu et al. [29] noticed a superior osseointegration and gene expression in these coated manufactured implants [29]. Xiu et al. [32] noticed early osteogenesis osseointegration, due to improvements in cellular functions and the higher protein content found [32]. Finally, Yin et al. [34] noticed resulted in greater uptake of plasma proteins, differentiation and adhesion of osteogenic cells that accelerated the osseointegration process [34].

## 4 Discussion

This systematic review addressed studies that evaluated the influence of surface treatments on titanium dental implants produced by AM on osseointegration, in the search to elucidate whether these treatments can maximize bone-implant contact and stimulate adhesion and absorption of related proteins. To this phenomenon. The research included contradicted the null hypothesis and indicated that, even in implants produced by additive manufacturing, surface treatments remain essential for the osseointegration process [4,5,9,11,12,[21], [22], [23], [24],[26], [27], [28], [29], [30],[32], [33], [34]].

In the selected articles, there was a great variety in the methodologies of surface treatments addressed that confuse the scientific and clinical community about the real need for these in titanium implants produced by AM since for each type of treatment, different topographic micro characteristics can be achieved and thus different results to osseointegration can be achieved [4,5,9,11,12,[21], [22], [23], [24],[26], [27], [28], [29], [30],[32], [33], [34]].

Several substances and techniques for performing surface treatments are found in the literature, being described as mechanical, such as machining and abrasive blasting, chemical, such as oxidation and acid conditioning, and physical, such as plasma spray [35]. Despite being different techniques, they all revolve around a central objective: to change the microtopographic properties of the surface and favor the biological phenomena that result in osseointegration.

The studies addressed in this review used different surface treatment techniques on implants produced by additive manufacturing to evaluate osseointegration [4,5,9,11,12,[21], [22], [23], [24],[26], [27], [28], [29], [30],[32], [33], [34]]. Regardless of the technique used, it was possible to infer that the surface treatment is of fundamental importance in the osseointegration process since it allows the creation of a micro-nano-textured structure that makes the surface more hydrophilic and allows better bone-implant contact [34] and that drives an improvement in the osteoconductive properties of the surface that results in the acceleration of adhesion and absorption of bone cells and proteins involved in the osseointegration process and bone neoformation. In addition, it is noted that the application of surface treatment improves the amount of residual particles on the surface, hinders adhesion and biofilm formation, in addition to boosting mechanical properties [9,11,27]. Performing surface treatments represent an effective strategy to reduce the risk of post-surgical inflammatory reactions [27] and thus results in a faster and more uniform bone growth pattern around the entire implant, Xiu et al. [32] since the surface finish allows a superior gene expression when compared to implants without treatment [32].

From these findings observed in the articles included and mentioned above, it is possible to perceive that the surface treatment, regardless of the technique or substance used, produces a more favorable surface for osseointegration, making it necessary even in implants produced by additive manufacturing. However, for this osseointegration phenomenon to occur, it is necessary to understand that it does not depend only on the natural biological behavior of the host, but also on the microstructure and chemical composition of the implant surface [32,36].

When considering the current state of the art regarding the additive manufacturing technique for the manufacture of titanium implants, it is noted the promotion of good macrostructural regulation, such as the control of porosity on the surface, which allows for better vascularization, formation of anchorage to bone tissue, differentiation and growth of osteoblastic cells that favor bone fixation [9,10,[26], [27], [28]], however, the implants produced by this technique still seems to need greater control of the microstructural characteristics responsible for the biological response and osseointegration. Failure to carry out the surface treatment, for example, can facilitate the release of dust particles that are not fused or poorly adhered to the implant surface at the implantation sites and increase the risk of local inflammatory processes or even induce failure points in the implant [1,23,32]. Therefore, several surface modifications are being studied in titanium dental implants produced by additive manufacturing as evaluated in the included articles, to improve their performance and osseointegration capacity [32].

The articles included do not present financial interests or personal relationships that constitute a conflict of interest and may have influenced the result of the work. In the construction of this manuscript, some limitations that made the review process difficult were found, the main one referring to the literary gap on the surface characteristics of titanium implants produced by additive manufacturing. In addition, a limitation in the evidence of the evaluated experimental studies was noted. However, it is possible to infer that the performance of surface treatments on these pieces are of fundamental importance to guarantee biological and microbiological responses that favor the osseointegration of these implants, thus better clinical responses will be obtained, which result in an acceleration of treatment completion and rehabilitative success, with greater patient satisfaction. Finally, the importance of more clinical and experimental studies on the subject is highlighted, with better standardization of methodology, reproducibility, and greater control for greater homogeneity in the results found.

## 5 Conclusion

Based on the results of this systematic review, it was possible to reach the following conclusions.

1. Although the additive manufacturing technique aims to produce surfaces with important characteristics and favorable to osseointegration, all included studies determined that the performance of surface treatment, regardless of the chosen technique, is necessary to bring about significant improvements in osseointegration;
2. The methodological heterogeneity of the surface treatments found in the included studies, represents the need to carry out more in-depth studies to determine which surface treatment, associated with additive manufacturing, can offer better results to osseointegration.

## Author contribution statement

João Vicente Calazans Neto: Conceived and designed the experiments; Performed the experiments; Analyzed and interpreted the data; Contributed reagents, materials, analysis tools or data; Wrote the paper.

Andréa Cândido dos Reis and Mariana Lima da Costa Valente Conceived and designed the experiments; Analyzed and interpreted the data; Contributed reagents, materials, analysis tools or data; Wrote the paper.

## Data availability statement

Data included in article/supplementary material/referenced in article.

## Declaration of competing interest

The authors declare that they have no known competing financial interests or personal relationships that could have appeared to influence the work reported in this paper.
