# Supplementary material for: Osseointegration in additive-manufactured titanium implants: A systematic review of animal studies on the need for surface treatment
Source: Heliyon. 2023 Jun 10;9(6):e17105. doi: 10.1016/j.heliyon.2023.e17105 (PMC10361303; doi:10.1016/j.heliyon.2023.e17105)
Supplement: Multimedia component 1 [file mmc1.docx]

SUPPLEMENTARY TABLE

Supplementary table 1 - Database search strategy.

| Database | Search | Found |
| --- | --- | --- |
| EMBASE  March  23^th,^ 2022 | ('titanium implants' OR 'titanium') AND ('additive manufacturing' OR '3d printing' OR 'electron beam melting' OR 'selective laser melting') AND ('surface modification' OR 'surface treatment') AND osseointegration | 33 |
| PubMed  March  23^th,^ 2022 | ('titanium implants' OR 'titanium') AND ('additive manufacturing' OR '3d printing' OR 'electron beam melting' OR 'selective laser melting') AND ('surface modification' OR 'surface treatment') AND osseointegration | 85 |
| Scopus  March  23^th,^ 2022 | ('titanium implants' OR 'titanium') AND ('additive manufacturing' OR '3d printing' OR 'electron beam melting' OR 'selective laser melting') AND ('surface modification' OR 'surface treatment') AND osseointegration | 2 |
| Science Direct  March  23^th,^ 2022 | ('titanium implants' OR 'titanium') AND ('additive manufacturing' OR '3d printing' OR 'electron beam melting' OR 'selective laser melting') AND ('surface modification' OR 'surface treatment') AND osseointegration | 790 |
| Google Scholar  January  16^th,^ 2023 | 'titanium implants' AND ('additive manufacturing' OR '3D printing' OR 'electron beam melting' OR 'selective laser melting') AND ('surface modification' OR 'surface treatment') AND (osseointegration OR 'osseo neoformation') | 93 |

Supplementary table 2- Risk of bias

| Autor/  ano | Was the allocation sequence adequately generated and applied? | Were the groups similar at baseline? | Was the allocation to the different groups adequately concealed during? | Were the animals randomly housed during the experiment? | Were the caregivers and/or investigators blinded from knowledge which intervention each animal received during the experiment? | Were animals selected at random for outcome assessment? | Was the outcome assessor blinded? | Were incomplete outcome data adequately addressed? | Are reports of the study free of selective outcome reporting? | Was the study apparently free of other problems that could result in high risk of bias? |
| --- | --- | --- | --- | --- | --- | --- | --- | --- | --- | --- |
| Bandyo  padhyay et  al., 2016 | YES | YES | YES | YES | UNCLEAR | YES | UNCLEAR | YES | YES | YES |
| Brogini  et al.,  2021 | YES | YES | YES | YES | YES | YES | YES | YES | YES | YES |
| Duan  et al.,  2020 | YES | YES | YES | YES | YES | YES | YES | YES | YES | YES |
| Gu et al., 2022 | UNCLEAR | YES | YES | YES | YES | YES | YES | YES | YES | YES |
| Huang et  al., 2020 | YES | YES | YES | YES | YES | YES | YES | YES | YES | YES |
| Lee et al., 2021 | UNCLEAR | YES | YES | YES | YES | YES | YES | YES | YES | YES |
| Li et al.,  2015 | UNCLEAR | YES | YES | YES | YES | YES | YES | YES | YES | YES |
| Lyu et al., 2020 | YES | YES | YES | YES | YES | YES | YES | YES | YES | YES |
| Mitra  et al.,  2021 | UNCLEAR | YES | YES | YES | YES | YES | YES | YES | YES | YES |
| Ren et al., 2020 | UNCLEAR | YES | YES | YES | YES | YES | YES | YES | YES | YES |
| Ronya  et al.,  2021 | YES | YES | YES | YES | YES | YES | YES | YES | YES | YES |
| Shu et al., 2020 | YES | YES | YES | YES | YES | YES | YES | YES | YES | YES |
| Tenga  et al.,  2019 | YES | YES | YES | YES | UNCLEAR | YES | UNCLEAR | YES | YES | YES |
| Wang  et al.,  2016 | UNCLEAR | YES | YES | YES | UNCLEAR | YES | UNCLEAR | YES | YES | YES |
| Wang  et al.,  2018 | UNCLEAR | YES | YES | YES | UNCLEAR | YES | UNCLEAR | YES | YES | YES |
| Xiu et al., 2016 | YES | YES | YES | YES | YES | YES | YES | YES | YES | YES |
| Xu et al., 2016 | YES | YES | YES | YES | UNCLEAR | YES | UNCLEAR | YES | YES | YES |
| Yavari  et al.,  2019 | YES | YES | YES | YES | UNCLEAR | YES | UNCLEAR | YES | YES | YES |
| Yin et al., 2021 | YES | YES | YES | YES | YES | YES | YES | YES | YES | YES |

Supplementary table 3- GRADE table of evidence for the effect of surface treatments on osseointegration of additive manufacturing titanium implants.

| **Outcome** | **№ of participants (studies)**  **Follow-up** | **Certainty of the evidence (GRADE)** | **Relative Effect (95% CI)** | **Potential absolute effects** | |
| --- | --- | --- | --- | --- | --- |
|  |  |  |  | **Surface treatment** | **Risk difference with surface treatment** |
| Osseointegration | 381 (17 EPCRs) | ⨁⨁⨁⨁ High | Not estimable | 57 per 100 | **57 minus for 100**  **(57 minus to 57 minus)** |
| **CI:** Confidence interval; | | | | | |
| **GRADE Working Group grades of evidence** **High certainty:** we are very confident that the true effect lies close to that of the estimate of the effect. **Moderate certainty:** we are moderately confident in the effect estimate: the true effect is likely to be close to the estimate of the effect, but there is a possibility that it is substantially different. **Low certainty:** our confidence in the effect estimate is limited: the true effect may be substantially different from the estimate of the effect. **Very low certainty:** we have very little confidence in the effect estimate: the true effect is likely to be substantially different from the estimate of effect. | | | | | |
